# Claudin Loss-of-Function Disrupts Tight Junctions and Impairs Amelogenesis

**DOI:** 10.3389/fphys.2017.00326

**Published:** 2017-05-24

**Authors:** Claire Bardet, Sandy Ribes, Yong Wu, Mamadou Tidiane Diallo, Benjamin Salmon, Tilman Breiderhoff, Pascal Houillier, Dominik Müller, Catherine Chaussain

**Affiliations:** ^1^Laboratory Orofacial Pathologies, Imaging and Biotherapies, Dental School, Paris Descartes University, Sorbonne Paris Cité Paris, France; ^2^Shanghai Key Laboratory of Stomatology, Department of Oral and Cranio-maxillofacial Science, Ninth People's Hospital, Shanghai Jiao Tong University School of Medicine Shanghai, China; ^3^Department of Odontology, AP-HP, and Reference Center for Rare Dieases of the Metabolism of Calcium and Phosphorus, Nord Val de Seine Hospital (Bretonneau) Paris, France; ^4^Department of Pediatric Nephrology, Charité University School of Medicine Berlin, Germany; ^5^Cordeliers Research Center, Centre National de la Recherche Scientifique, Institut National de la Santé et de la Recherche Médicale UMRS 1138, Paris-Diderot, Pierre et Marie Curie and Paris Descartes Universities, ERL Paris, France

**Keywords:** *Amelogenesis Imperfecta*, enamel, barrier-forming tight junction protein, pore-forming tight junction protein, claudins

## Abstract

Claudins are a family of proteins that forms paracellular barriers and pores determining tight junctions (TJ) permeability. Claudin-16 and -19 are pore forming TJ proteins allowing calcium and magnesium reabsorption in the thick ascending limb of Henle's loop (TAL). Loss-of-function mutations in the encoding genes, initially identified to cause Familial Hypomagnesemia with Hypercalciuria and Nephrocalcinosis (FHHNC), were recently shown to be also involved in *Amelogenesis Imperfecta* (AI). In addition, both claudins were expressed in the murine tooth germ and *Claudin-16* knockout (KO) mice displayed abnormal enamel formation. Claudin-3, an ubiquitous claudin expressed in epithelia including kidney, acts as a barrier-forming tight junction protein. We determined that, similarly to claudin-16 and claudin-19, claudin-3 was expressed in the tooth germ, more precisely in the TJ located at the apical end of secretory ameloblasts. The observation of *Claudin-3* KO teeth revealed enamel defects associated to impaired TJ structure at the secretory ends of ameloblasts and accumulation of matrix proteins in the forming enamel. Thus, claudin-3 protein loss-of-function disturbs amelogenesis similarly to claudin-16 loss-of-function, highlighting the importance of claudin proteins for the TJ structure. These findings unravel that loss-of-function of either pore or barrier-forming TJ proteins leads to enamel defects. Hence, the major structural function of claudin proteins appears essential for amelogenesis.

Epithelial cells are attached to each other at their lateral membranes by a complex of intercellular junctions (Farquhar and Palade, 1963). The most apical complex of the intercellular junctions is the *zona occludens* also named tight junctions (TJ). TJ represent the principal component of the paracellular diffusion barrier by determining epithelial permeability of small molecules and water, and participate in the control of the diffusion of membrane components between the basolateral and apical regions. Abrogation of such a barrier function in epithelia that interfaces with the environment is associated with a variety of gastrointestinal (Barmeyer et al., 2017), renal (Hou, 2014), and cutaneous disorders (Basler and Brandner, 2017). TJ are composed of several transmembrane and membrane-associated proteins including the claudin proteins. Claudins form either paracellular barrier or pores that determine TJ properties. They interact with each other and with additional membrane and non-membrane proteins, such as the intracellular *zonula occludens* ZO-1 and ZO-2 and other PDZ domain-containing proteins. Claudins are considered as core components of TJ strands and determine epithelial permeability of small molecules and water (Gunzel and Yu, 2013). Only few claudins are unequivocally qualified as pore-forming proteins, including claudin-2, -10b, and -15 as cation pores and claudin-10a and -17 as anion pores. Other claudins have been reported to form pores only when specifically interacting with another claudin (Gunzel and Yu, 2013). Such is the case of claudin-16 and claudin-19, which form a cation-selective TJ complex in the thick ascending limb of Henle's loop (Hou et al., 2008).

Mineral transport involves the epithelial permeability which is tightly related to the type and properties of TJ. During amelogenesis, secretory ameloblast TJ are responsible for restricted paracellular access to the enamel compartment. The paracellular permeability (tightness) of the ameloblast layer depends on the composition of TJ relying on claudin proteins. Hence, a combination of different claudins may either allow some paracellular ion passage or restrict this passage (Denker and Sabath, 2011; Bronckers, 2017). To date, 11 claudins have been identified in the tooth germ at various developmental stages (Ohazama and Sharpe, 2007; Bardet et al., 2016; Yamaguti et al., 2017). We recently associated the abrogation of a pore function and a dental disorder. Indeed, we demonstrated that loss-of-function mutations in *Claudin-16 and -19* (*CLDN16* and *19*) genes, initially identified to cause Familial Hypomagnesemia with Hypercalciuria and Nephrocalcinosis (FHHNC), also resulted in *Amelogenesis Imperfecta (AI)* (Bardet et al., 2016; Yamaguti et al., 2017). At the time of this discovery, it was acknowledged that claudin-16 and -19 were mainly expressed in the thick ascending limb of Henle loop in the kidney, suggesting that the *AI* diagnosed in patients with FHHNC was a consequence of the disturbed mineral homeostasis. However, we were able to show that claudin-16 and claudin-19 were also expressed in the ameloblast TJ, indicating that the *AI* diagnosed in patients with FHHNC was an intrinsic consequence of the *Claudin* mutation. Furthermore, studying *Claudin-16* Knockout (KO) mice, we showed that the structure of ameloblast TJ was altered.

Claudin-3, which is expressed in epithelia of a wide variety of organs such as intestine, kidney, liver, skin, and lung, acts as a barrier-forming TJ protein (Milatz et al., 2010; Gunzel and Yu, 2013). To analyze the substantial role of a barrier-forming TJ protein in amelogenesis, we explored the dental phenotype of *Claudin-3* KO mice using the methods previously described in Bardet et al. (2016) (ethical agreement D92-049-01). We first determined that, similar to claudin-16 and claudin-19 (Bardet et al., 2016; Yamaguti et al., 2017), claudin-3 was expressed in the tooth germ and located in the TJ at the apical end of secretory ameloblasts (Figures 1A,B). Dental examination of adult *Claudin-3* KO mice revealed a lower enamel volume in molars when compared to WT mice and a mineralization delay in the continuously growing incisor (Figure 1C). At the structural level, when examining the forming enamel in the intrabony part of the incisor, we observed normally formed prisms by scanning electron microscopy in *Claudin-3* KO mice (Figure 1D). However, transmission electron microscopy (TEM) analysis of tooth germs showed altered TJ structure in *Claudin-3* KO ameloblasts displaying thicker and packed structures compared to the thin intermediate line observed in the WT mice (Figure 1D). At the molecular level, enamel matrix formation was disturbed by claudin-3 deficiency, manifesting by an accumulation of enamel proteins further confirmed by Western blot analysis of the protein extracts of the soft part of the growing incisor (Figure 1E). Thus, claudin-3 protein loss-of-function disturbs amelogenesis similar to claudin-16 loss-of-function, where accumulation of enamel matrix protein led to *Amelogenesis Imperfecta* (Bardet et al., 2016).

**Figure 1:**
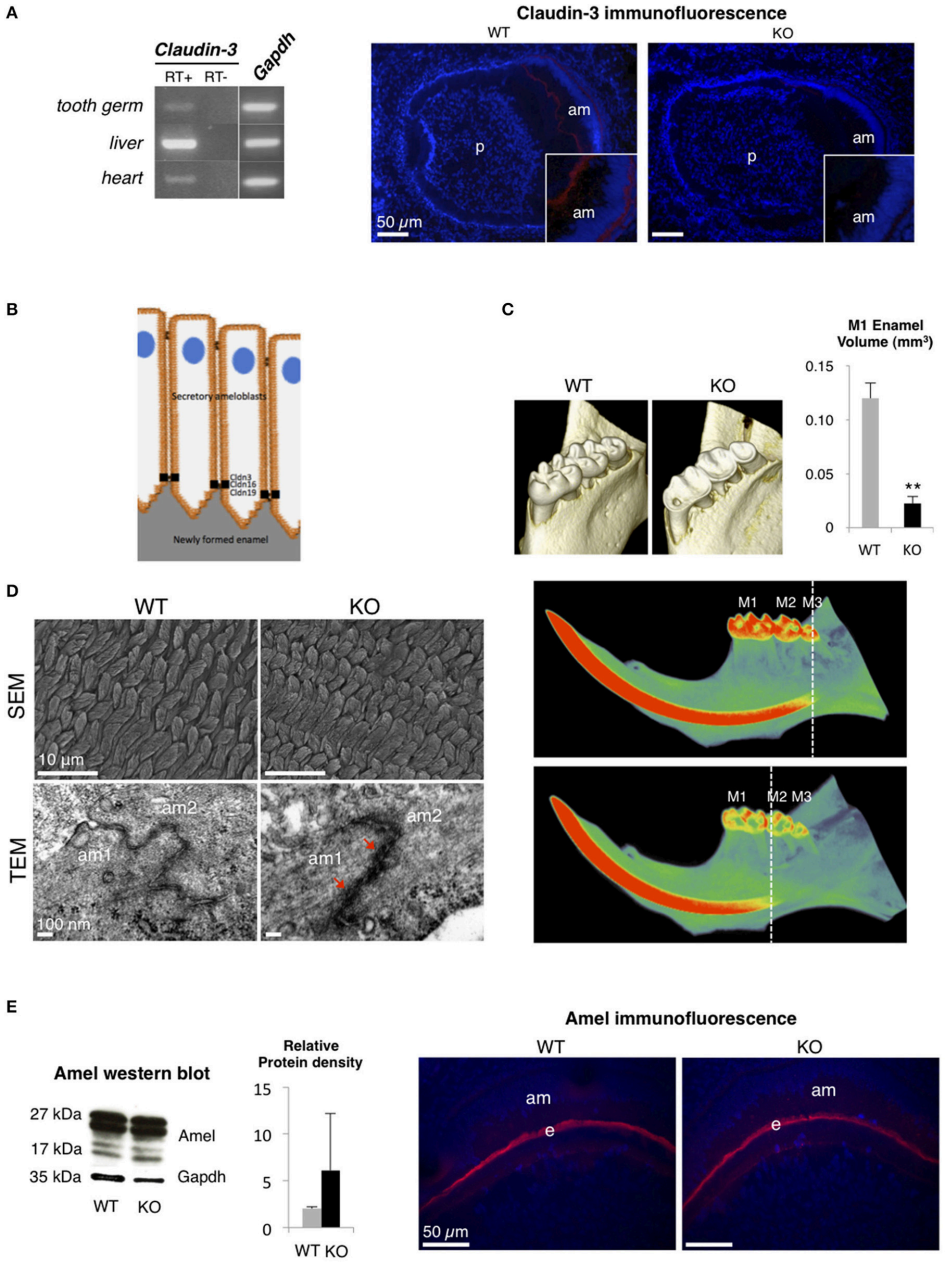
**Dental phenotype of ***Claudin-3*** KO mice. (A)** *Claudin-3* mRNA expression was analyzed in mouse tissues by RT-PCR using forward primer 5′-ATG TGG CGC GTT TCG-3′ and reverse primer 5′-GCG AGT CGT ACA TTT TGC-3′ for *Claudin-3* (124 bp), and forward primer 5′-TGT GTC CGT CGT GGA TCT GA-3′ and reverse primer 5′-TTG CTG TTG AAG TCG CAG GAG-3′ for *Gapdh* (150 bp). A representative gel image of the selected tissues showed *Claudin-3* expression in mouse in post-natal day 3 tooth germ, liver and heart. Identification of claudin-3 in the continuously growing incisor was performed by immunofluorescence using claudin-3 antibody (#34-1700, Invitrogen) at 1/100 dilution and a Goat anti-Rabbit antibody (#R-6394, Invitrogen). Claudin-3 was localized at the distal end of secretory ameloblasts (am). Protein expression was also observed at the basal end of the cells (*n* = 3 per group). (p) pulp. **(B)** Recapitulative schema of claudin TJ proteins expression at the apical end of secretory ameloblasts. Claudin-3, -16, and -19 were shown to be expressed at TJ level of ameloblast secretory ends during enamel formation in the mouse. **(C)** 3D volume rendering from Micro-CT data showed severe enamel loss on lingual molar cusps in *Claudin-3* KO mice. Quantitative analysis confirmed a significant lower enamel volume in *Claudin-3* KO mice when compared to WT (0.022 vs. 0.120 mm^3^ respectively) (*n* = 8 per group). In WT mice, incisor mineralization is observed under the third molar (M3) whereas in *Claudin-3* KO mice it is detected under the second molar (M2). ^**^P < 0.00001 (M1) first molar. **(D)** Scanning electron microscopy (SEM) analysis showed enamel prisms normally constituted in *Claudin-3* KO incisor when compared to WT (*n* = 3 per group). Transmission electron microscopy (TEM) analysis of WT tooth germs showed TJ as adjoining ameloblast membranes converging to form a thin intermediate line at the distal end of the cells. In contrast, the TJ structure was altered in *Claudin-3* KO tooth germs, with thicker and packed structures (red arrows). (am) ameloblast. **(E)** Enamel matrix protein expression by secretory ameloblasts of *Claudin-3* KO mice (*n* = 3 per group). Western blot analysis of the protein extracts of the soft part of the growing incisor showed slightly higher levels of amelogenin (amel). No difference was observed by immunostaining regarding amelogenin expression in the forming enamel matrix (e) between *Claudin-3* KO and WT incisors.

During amelogenesis, TJ are composed of pore- and barrier-forming claudin proteins. Deficiency of both types of claudin proteins in murine models as well as in human disorders such as FHHNC led to enamel defects. To date, no human disease was associated with *CLDN3* mutation. Interestingly, the hemizygous contiguous gene microdeletion at 7q11.23 in Williams-Beuren syndrome (WBS; OMIM 194050) includes the *CLDN3* gene (Dutra et al., 2011) and several dental manifestations have been reported in patients with WBS, including hypodontia, abnormal tooth shape (microdontia), but also hypoplastic enamel defects and higher caries susceptibility (Hertzberg et al., 1994; Axelsson et al., 2003).

Our observations highlight the importance of claudin proteins for TJ structure. Indeed, either barrier or a pore forming claudins link to adaptor proteins such as ZO proteins and other PDZ protein family of the cytosolic TJ plaque. This interaction allows direct or indirect bonding to actin, anchoring the TJ within the underlying cytoskeleton. This scaffold facilitates the assembly of highly ordered structures, such as junctional complexes, regulating epithelial cell polarity, proliferation and differentiation (Sluysmans et al., 2017). This is consistent with our data showing a disturbed actin filament network at apical end of ameloblasts and a more diffused ZO-1 labeling in *Claudin-16* KO mice (Bardet et al., 2016). Claudins are necessary for the TJ assembly process and their loss impairs TJ organization in ameloblasts and consequently disturbs enamel formation.

Overall, although TJ insure suitable microenvironments for enamel deposition and concomitant early maturation by determining the paracellular permeability and selectivity of solutes, the ion transport is tightly regulated during amelogenesis, involving barrier or channel properties of a certain claudin. Impaired TJ structure resulting from claudin loss-of function disturbs enamel formation. Hence, the major structural function of claudin proteins appears essential for amelogenesis.

## Author contributions

CB and CC wrote the manuscript with contributions from all authors. CB, SR, YW, and CC contributed to the design of the experiments. CB, SR, YW, MD, BS, TB, PH, DM, and CC performed and analyzed experiments. CB and CC supervised the project. All authors reviewed and approved the final version of the manuscript.

### Conflict of interest statement

The authors declare that the research was conducted in the absence of any commercial or financial relationships that could be construed as a potential conflict of interest. The reviewer YZ and handling Editor declared their shared affiliation, and the handling Editor states that the process nevertheless met the standards of a fair and objective review.
